# Impact of an infectious disease specialist-led antimicrobial stewardship program and consultations during multiple antimicrobial shortages: A bayesian structural time-series analysis

**DOI:** 10.1371/journal.pone.0340599

**Published:** 2026-02-02

**Authors:** Naoya Itoh, Nobumasa Okumura, Nana Akazawa-Kai, Chiharu Wachino, Shunsuke Kuriki, Takanori Kawabata

**Affiliations:** 1 Department of Infectious Diseases, Graduate School of Medical Sciences, Nagoya City University, Aichi, Japan; 2 Nagoya City University East Medical Center, Nagoya, Japan; 3 Department of Clinical Pharmaceutics, Graduate School of Medical Sciences, Nagoya City University, Aichi, Japan; 4 Department of Pharmacy, Nagoya City University East Medical Center, Aichi, Japan; 5 Department of Data Science, National Cerebral and Cardiovascular Center, Suita, Japan; Fayetteville State University, UNITED STATES OF AMERICA

## Abstract

Since antimicrobial shortages are becoming common owing to manufacturing issues and sudden demand, individualized antimicrobial optimization by infectious disease specialists is essential. We evaluated the effects of a 12-month antimicrobial stewardship program at a university-affiliated hospital in Japan during antimicrobial shortages. In this single-center retrospective observational study, from April 1, 2023, to March 31, 2025, we compared the pre-intervention (antimicrobial stewardship program without infectious disease specialists) and post-intervention (antimicrobial stewardship program with infectious disease specialists and formal consultation service) periods. The evaluated outcomes included antimicrobial use, microbiological indicators (number of culture specimens submitted and hospital-acquired resistant organisms), patient outcomes (in-hospital mortality and length of hospital stay), and antimicrobial costs. A Bayesian structural time-series analysis adjusted for seasonality was used to assess intervention effects. In total, 24,601 inpatients were included (11,782 before intervention and 12,819 after). Shortages affected nine intravenous and two oral antibiotics. The antimicrobial stewardship program team provided 1110 feedback instances (acceptance rate, 74.1%) and conducted 172 infectious disease consultations in the post-intervention period. Carbapenem use, antipseudomonal agent use, incidence of carbapenem-resistant *Pseudomonas aeruginosa*, and the cost of carbapenems per patient-day significantly decreased. Narrow-spectrum antibiotic use, anti-methicillin-resistant *Staphylococcus aureus* agent use, all intravenous antimicrobial use, total intravenous and oral antimicrobials, and the number of inpatient specimens were significantly increased. All antimicrobial costs, in-hospital mortality, and length of hospital stay remained unchanged. Antimicrobial stewardship program supported by infectious disease specialists/consultants significantly reduced carbapenem and antipseudomonal agent use without negatively affecting patient outcomes. These findings highlight infectious disease specialists’ critical role in supporting effective antimicrobial stewardship programs, particularly during limited antimicrobial supply and increased clinical complexity.

## Introduction

In recent years, antimicrobial shortages have become increasingly frequent. This is due to noncompliance with good manufacturing practices; regulatory delays; unavailability of essential components, including active pharmaceutical ingredients; unexpected surges in demand; and increased misuse or overuse of antimicrobials, which may accelerate depletion of limited drug supplies [1]. Antimicrobial shortages pose substantial challenges to patient care, public health, and antimicrobial stewardship (AS) efforts worldwide [1,2].

Recent systematic reviews have provided evidence that antimicrobial shortages are a worldwide issue affecting high-income and low- and middle-income countries [3]. Shortages have most frequently been reported for piperacillin/tazobactam, penicillin G, gentamicin, and meropenem, which are essential for treating severe bacterial infections. Antimicrobial shortages lead to prolonged hospital stays, treatment failures following the use of inferior alternative antimicrobial therapies, increased adverse drug events, worsened patient outcomes, emergence of antimicrobial-resistant organisms, and detrimental effects on antimicrobial stewardship [3,4].

In Japan, a large-scale shortage of cefazolin was reported in 2019 [2,5]. During this period, many healthcare facilities were required to revise their antimicrobial use policies and adopt alternative agents. This consequently led to increased use of broad-spectrum antibiotics, higher healthcare costs, and heightened concerns regarding antimicrobial resistance [2].

To mitigate their impact, healthcare facilities must optimize antimicrobial selection and minimize disruptions. They also should implement robust antimicrobial stewardship programs (ASPs), which are coordinated interventions for improving and measuring appropriate use of antimicrobial agents, by promoting optimal drug regimen selection, including dosing, therapy duration, and route of administration [6,7]. During antimicrobial shortages, ASPs have been reported to contribute to the effective conservation of antimicrobials through monitoring of drug inventories, audits of antimicrobial use, and by providing guidance on alternative therapies of agents in limited supply [8]. As part of ASPs, requiring prior approval by an infectious disease (ID) specialist is another approach used to ensure appropriate antimicrobial use during antimicrobial shortages [9]. Amid ongoing antibiotic shortages, the optimization of patient-specific antimicrobial therapy, facilitated by ID specialists with extensive and adaptable expertise, is of paramount importance. Therefore, we evaluated the impact of an ID specialist-led 12-month ASP intervention and ID consultations in a Japanese university-affiliated hospital experiencing multiple antimicrobial shortages.

## Patients and methods

### Ethics

The study protocol, approved by the Institutional Review Board (IRB) of the Nagoya City University (approval number: 60-24-0023), was conducted in accordance with the principles of the Declaration of Helsinki. The requirement for informed consent was waived by the IRB because only data collected in clinical practice were used.

### Study design and setting

This study was conducted at Nagoya City University East Medical Center (NCUEMC), Aichi, Japan, a 498-bed tertiary care facility. The center comprises 30 clinical departments and admits approximately 10,000 patients annually. This single-center, retrospective observational study included data obtained from the NCUEMC database, including microbiology profiles from the microbiology laboratory, prescription data from the pharmacy department, patient data from AS team (AST) conferences, and medical records, from April 1, 2023, to March 31, 2025. Data were accessed for research purposes on April 1, 2025. This study included the data of all patients who were hospitalized during the study period.

### Restrictions on the supply of antimicrobials

During the study period, the supply of nine intravenous antibiotics and two oral antibiotics was restricted. The focus was carbapenems (CARs) because of their broad-spectrum activity, highlighting the importance of their appropriate use. Among CARs, imipenem/cilastatin was usage-restricted from December 19, 2022, to March 1, 2024, and meropenem since February 1, 2023. “Usage-restricted” refers to the absence of system-based ordering restrictions, with prescribers advised to adjust prescriptions on a case-by-case basis in response to stock shortages. Imipenem/cilastatin and doripenem were order-stopped from March 1, 2024, and December 14, 2024, respectively. “Order-stopped” refers to the complete suspension of prescription orders, allowing usage only for specific patients under special circumstances. Details regarding supply restrictions for other antibiotics are provided in Table 1.

**Table 1:** Restriction of antibiotic supply during the study period.

Antimicrobials	Category	Restriction start date (Y/M/D)	Restriction end date (Y/M/D)	Restriction type
**IPM/CS**	Injection	12/19/2022	3/1/2024	Usage-restricted^a^
**MEPM**	Injection	2/1/2023	Ongoing	Usage-restricted^a^
**CPZ/SBT**	Injection	3/24/2023	8/30/2023	Order-stopped^b^
**AMPC/CVA**	Tablet	8/22/2023	10/28/2023	Order-stopped^b^
**CMZ**	Injection	8/24/2023	Ongoing	Usage-restricted^a^
**MINO**	Injection	10/31/2023	Ongoing	Order-stopped^b^
**CTM**	Injection	12/8/2023	2/19/2025	Order-stopped^b^
**IPM/CS**	Injection	3/1/2024	10/2/2024	Order-stopped^b^
**PIPC**	Injection	4/6/2024	8/1/2024	Order-stopped^b^
**AMPC/CVA**	Tablet	5/31/2024	8/3/2024	Order-stopped^b^
**CPZ/SBT**	Injection	6/28/2024	Ongoing	Order-stopped^b^
**CEX**	Capsule	9/4/2024	Ongoing	Order-stopped^b^
**AMPC/CVA**	Tablet	10/25/2024	Ongoing	Order-stopped^b^
**DRPM**	Injection	12/14/2024	Ongoing	Order-stopped^b^
**EM**	Injection	12/17/2024	Ongoing	Order-stopped^b^

Abbreviations: IPM/CS, imipenem/cilastatin; MEPM, meropenem; CPZ/SBT, cefoperazone/sulbactam; AMPC/CVA, amoxicillin/clavulanate; CMZ, cefmetazole; MINO, minocycline; CTM, cefotiam; PIPC, piperacillin; CEX, cefalexin; DRPM, doripenem; EM, erythromycin

^a^Usage-restricted refers to situations in which no restrictions are implemented within the ordering system, although prescribers are advised to modify prescriptions on a case-by-case basis due to stock shortages.

^b^Order-stopped refers to the complete suspension of prescription orders, with use permitted only for specific patients under special circumstances.

### Interventions

1)
**Pre-intervention period (April 1, 2023, to March 31, 2024):** An AST conference was held once a week to conduct retrospective audits and provide feedback on inpatients who received the designated antimicrobial agents for > 1 week. The designated antimicrobials included CARs (imipenem/cilastatin, meropenem, and doripenem); antipseudomonal agents (cefepime, piperacillin/tazobactam, and ceftolozane/tazobactam); and anti-methicillin-resistant *Staphylococcus aureus* (MRSA) agents (vancomycin, teicoplanin, daptomycin, and linezolid). The AST responsible for the ASP comprised one non-ID physician, pharmacist, nurse, and laboratory technician each.
2)
**Post-intervention period (April 1, 2024, to March 31, 2025):** The ASP supported by ID specialists and ID consultation (defined as a direct request by the primary care team to ID specialists for diagnosis or treatment assistance, independent of the ASP intervention) was initiated. The ASP intervention, supported by three ID specialists (post-graduate clinical experience, 11–18 years), included the following: (1) on weekdays, ID specialists communicated positive blood culture results to the primary care team to facilitate timely initiation of appropriate empirical therapy. (2) Audits were conducted three times a week for all hospitalized patients receiving CARs, regardless of the duration of use. For anti-MRSA and antipseudomonal agents administered for ≥ 7 days (after ≥ 3 days for antipseudomonal agents from October 2024), audits were conducted. From December 2024, audit frequency covered all weekdays. During AST conferences, ID specialists provided educational feedback and pharmacists gave specific recommendations to attending physician teams on all cases reviewed. Cases already under ID consultation were excluded from the audit (see S1 File).

### Outcome measures

The primary study outcome was change in the number of days of therapy (DOTs) with intravenous CARs (CAR-DOTs; including imipenem-cilastatin, meropenem, and doripenem), calculated as DOTs per 100 patient-days per month. Secondary outcomes included DOTs for three antipseudomonal agents (piperacillin-tazobactam, cefepime, and ceftolozane-tazobactam); six narrow-spectrum antimicrobials (penicillin G, ampicillin, ampicillin/sulbactam, cefazolin, cefmetazole, and flomoxef); and four anti-MRSA agents (vancomycin, teicoplanin, daptomycin, and linezolid). Additionally, DOTs were evaluated for all antimicrobials targeted for intervention, all intravenous antimicrobials, all oral antimicrobials, and the total combined intravenous and oral agents. For the combined therapy, each agent administered on the same day contributed one DOT. Other evaluated parameters included hospital-acquired resistant organisms and *Clostridioides difficile* infections (CDIs) incidence rates; CARs and all antimicrobials’ costs; number of culture samples collected per 1000 patient-days; all-cause in-hospital mortality rate; length of hospital stay; and assessment and acceptance rates of AST recommendations (details are provided in the S1 File) [10–13].

### Statistical analyses

From April 1, 2023, to March 31, 2025, we conducted a Bayesian structural time-series (BSTS) analysis using CausalImpact [14] in R software (version 4.2.0; R Foundation for Statistical Computing, Vienna, Austria) to assess the impact of the intervention on primary and secondary outcomes. The BSTS approach explicitly adjusted for trends and seasonality to provide counterfactual predictions and estimate what the outcomes would have been in the absence of the intervention. The model was specified as a univariate time-series model consisting of two components: (1) a local level component to capture the underlying baseline trend and (2) a seasonal component with a 12-month period to account for monthly seasonal fluctuations. No external covariates were included in the model; thus, the counterfactual prediction relied solely on the pre-intervention time-series history of the outcome variable. Model parameters were estimated via Markov chain Monte Carlo sampling with 1,000 iterations. The causal effect was summarized with posterior probabilities and 95% credible intervals (CrIs). This approach provided robust inference compared with non-Bayesian interrupted time-series methods. We implemented the t-test before-after change in purchase costs of CARs and all intravenous antimicrobials per patient-days from 2023 to 2025. All analyses were performed using R software (version 4.2.0). There were no missing data in our study.

## Results

### Study participants

Of 24,601 patients admitted during the study period, 11,782 (monthly average: 981.8 ± 14.6) and 12,819 (monthly average: 1068.3 ± 13.9) were in the pre-intervention and post-intervention phases, respectively. During the intervention phase, the AST provided 1110 instances of feedback on specific antimicrobials. There were 172 ID consultations throughout the intervention period.

### Appropriateness and acceptance of AST recommendations

Based on the AST evaluations, 494 appropriate (44.5%) and 616 inappropriate (55.5%) instances of antimicrobial use were determined. The overall acceptance rate of the 1110 AST suggestions was 74.1 ± 8.1%. Appropriate cultures were obtained before initiating designated antibacterial agents in 68.1 ± 7.6% of cases.

### Antibiotic consumption

#### Use of CARs.

**Fig 1:**
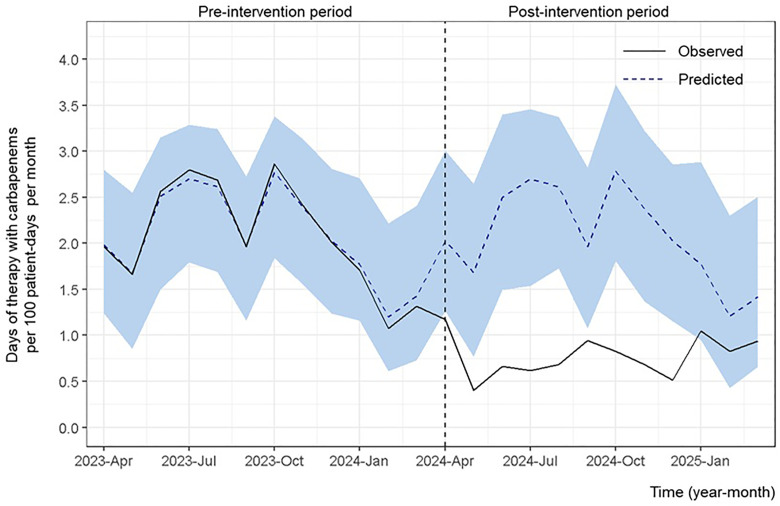
Days of therapy for carbapenems (imipenem/cilastatin, meropenem, and doripenem) per 100 patient-days per month. The days of therapy (DOTs) for the three carbapenems—imipenem/cilastatin, meropenem, and doripenem—per 100 patient-days per month are shown. The study had two phases: the pre-intervention (April 1, 2023, to March 31, 2024) and post-intervention (April 1, 2024, to March 31, 2025) periods. The light blue shaded area denotes the 95% credible interval around the predicted values. A significant reduction in DOTs for the three carbapenems is observed (average effect: −1.31; 95% credible interval: −1.55 to −1.10).

Fig 1 shows the changes in CAR-DOTs during the two phases. The DOTs significantly decreased for CARs (average effect: −1.31 [95% CrI, −1.55 to −1.10]).

#### Use of antipseudomonal agents.

**Fig 2:**
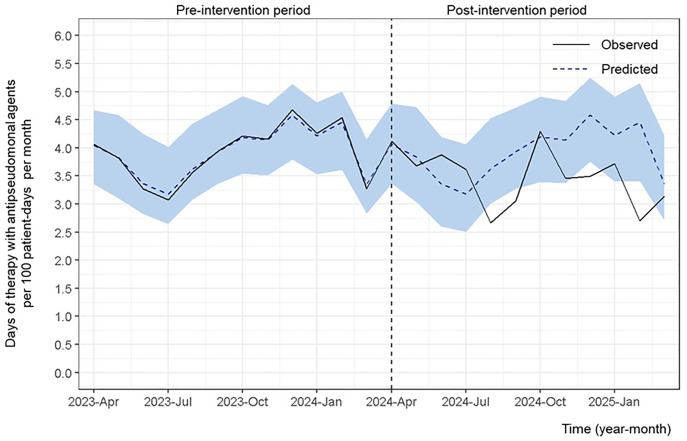
Days of therapy for antipseudomonal agents (cefepime, piperacillin/tazobactam, and ceftolozane/tazobactam) per 100 patient-days per month. The days of therapy (DOTs) for the three antipseudomonal agents—cefepime, piperacillin/tazobactam, and ceftolozane/tazobactam—per 100 patient-days per month are shown. The study had two phases: the pre-intervention (April 1, 2023, to March 31, 2024) and post-intervention (April 1, 2024, to March 31, 2025) periods. The light blue shaded area denotes the 95% credible interval around the predicted values. The DOTs are significantly decreased for the three antipseudomonal agents (average effect: −0.43; 95% credible interval, −0.80 to −0.08).

Fig 2 shows that the DOTs for the three antipseudomonal agents significantly decreased (average effect: −0.43; 95% CrI, −0.80 to −0.08).

#### Use of narrow-spectrum antibiotics.

The DOTs significantly increased for the six narrow-spectrum antibiotics (average effect: 2.64; 95% CrI, 2.22 to 3.06; Table 2).

**Table 2:** Bayesian structural time-series analysis of antimicrobial use, incidence of antimicrobial-resistant microorganisms, laboratory metrics, and patient outcomes.

	Pre-intervention mean	Post-intervention mean	Average effect	95% CrI
**Use of antimicrobials**				
**DOTs for carbapenems**^**a**^ **per 100 patient-days per month**	2.09	0.78	−1.31	−1.55, −1.10
**DOTs for antipseudomonal agents**^**b**^ **per 100 patient-days per month**	4.00	3.58	−0.43	−0.80, −0.08
**DOTs for narrow-spectrum antibiotics**^**c**^ **per 100 patient-days per month**	16.90	19.54	2.64	2.22, 3.06
**DOTs for anti-MRSA antimicrobials**^**d**^ **per 100 patient-days per month**	1.07	1.48	0.40	0.27, 0.52
**DOTs for all intravenous antimicrobials per 100 patient-days per month**	32.22	33.87	1.64	1.00, 2.29
**DOTs for all oral antimicrobials per 100 patient-days per month**	16.13	16.14	−0.01	−0.90, 0.72
**DOTs for total intravenous and oral antimicrobials per 100 patient-days per month**	48.36	50.01	1.63	0.10, 3.09
**Incidence of antimicrobial-resistant microorganisms**				
**ESBL-producing Enterobacterales per 1000 patient-days per month**	0.43	0.47	0.04	−0.06, 0.16
**CDI per 1000 patient-days per month**	0.10	0.12	0.02	−0.01, 0.04
**CRPA per 1000 patient-days per month**	0.10	0.05	−0.05	−0.08, −0.02
**MRSA per 1000 patient-days per month**	0.70	0.84	0.13	−0.02, 0.24
**Laboratory metrics**				
**Total cultures per 1000 patient-days**	32.44	38.77	6.33	5.23, 7.28
**Blood cultures per 1000 patient-days**	22.63	27.25	4.65	3.87, 5.55
**Respiratory cultures**^**e**^ **per 1000 patient-days**	6.26	7.39	1.12	0.81, 1.45
**Gastrointestinal cultures**^**f**^ **per 1000 patient-days**	2.03	2.28	0.24	0.11, 0.38
**Genitourinary cultures**^**g**^ **per 1000 patient-days**	4.85	6.14	1.31	0.97, 1.68
**Puncture fluid cultures**^**h**^ **per 1000 patient-days**	17.07	19.50	2.42	1.77, 3.07
**Other cultures**^**i**^ **per 1000 patient-days**	2.23	3.55	1.32	1.13, 1.53
**Patient outcomes**				
**All-cause mortality**	3.32	3.25	−0.08	−0.37, 0.14
**Average length of hospital stay**	12.12	11.86	−0.26	−0.65, 0.15

Abbreviations: CrI, credible interval; DOTs, days of therapy; ESBL, extended-spectrum β-lactamase; CDI, *Clostridioides difficile* infection; CRPA, carbapenem-resistant *Pseudomonas aeruginosa*; MRSA, methicillin-resistant *Staphylococcus aureus*.

^a^Imipenem/cilastatin, meropenem, and doripenem.

^b^Cefepime, piperacillin/tazobactam, and ceftolozane/tazobactam.

^c^Penicillin G, ampicillin, ampicillin/sulbactam, cefazolin, cefmetazole, and flomoxef.

^d^Vancomycin, teicoplanin, daptomycin, and linezolid.

^e^Respiratory specimens included sputum, pharyngeal mucus, nasal mucus, oral mucus, lung tissue, and bronchial lavage fluid.

^f^Gastrointestinal specimens encompassed stool, bile, and pancreatic fluid.

^g^Genitourinary specimens consisted of urine and vaginal secretions.

^h^Puncture fluid specimens comprised pleural, ascitic, spinal, joint, and bone marrow fluids.

^i^Other materials included catheter tips, wound cultures, drain cultures, and cultures obtained from various sources.

#### Use of anti-MRSA antimicrobials.

The DOTs significantly increased for the four anti-MRSA antimicrobials (average effect: 0.40; 95% CrI, 0.27 to 0.52; Table 2).

#### Use of all intravenous antimicrobials, all oral antimicrobials, and combined intravenous and oral antimicrobials.

The DOTs significantly increased for all intravenous antimicrobials (average effect: 1.64; 95% CrI, 1.00 to 2.29; Table 2). A significant increase was also observed in DOTs for the total intravenous and oral antimicrobials per 100 patient-days per month (average effect: 1.63; 95% CrI, 0.10 to 3.09). No significant change was observed in the total DOTs for all oral antimicrobials alone.

### Incidence of antimicrobial-resistant microorganisms and CDI

No cases of carbapenemase-producing *Enterobacteriaceae* were identified during the study period. The incidences of carbapenem-resistant *Enterobacteriaceae* were 0 ± 0 and 0.027 ± 0.04 per 1000 patient-days in the pre-intervention and post-intervention phases, respectively. However, due to the low event frequency, statistical analysis could not be conducted. A significant decrease was observed in the incidence of carbapenem-resistant *Pseudomonas aeruginosa* (CRPA), whereas no significant changes were noted in extended-spectrum β-lactamase–producing Enterobacterales, CDI, or MRSA (Table 2).

### Cost of CARs and all antimicrobials

CAR purchase cost per patient-day decreased from 3.60 USD (2023) to 1.49 USD (2024) significantly (p < 0.001). However, for all intravenous antimicrobials or both intravenous and oral antimicrobials, cost per patient-day did not change significantly (Table 3).

**Table 3:** Purchase costs of carbapenems and all intravenous antimicrobials per patient-days from 2023 to 2024.

Period	Cost of CARs per patient-days, USD	Cost of all intravenous antimicrobials per patient-days, USD	Cost of all oral antimicrobials per patient-days, USD	Total cost of all intravenous and oral antimicrobials per patient-days, USD
**Pre-intervention period (April 1, 2023, to March 31, 2024)**	3.60	46.17	1.28	47.45
**Post-intervention period (April 1, 2024, to March 31, 2025)**	1.49	48.53	1.17	49.70

Abbreviation: CAR, carbapenem

The cost calculation was based on an exchange rate of 150 yen per US dollar, as of March 2025.

### Culture specimen submissions

During the post-intervention period, the number of monthly cultures performed per 1000 patients increased significantly, with average effects of 6.33 (95% CrI, 5.23–7.28) for total cultures, 4.65 (95% CrI, 3.87–5.55) for blood cultures, 1.12 (95% CrI, 0.81–1.45) for respiratory cultures, 0.24 (95% CrI, 0.11–0.38) for gastrointestinal cultures, 1.31 (95% CrI, 0.97–1.68) for genitourinary cultures, 2.42 (95% CrI, 1.77–3.07) for puncture fluid cultures, and 1.32 (95% CrI, 1.13–1.53) for other cultures (Table 2).

### All-cause in-hospital mortality and length of hospital stay

No significant changes were observed in the all-cause in-hospital mortality rate or length of hospital stay (Table 2).

## Discussion

This study evaluated whether ASP supported by ID specialists and consultations optimized antimicrobial use during multiple antimicrobial shortages at a university-affiliated hospital in Japan. Despite these shortages, the intervention reduced the use of CARs, antipseudomonal agents, and incidence of CRPA, without adversely affecting patient outcomes.

During simultaneous shortages of multiple antimicrobial agents, our intervention was associated with a statistically significant reduction in CAR consumption. Importantly, this reduction did not coincide with an increase in the use of alternative antipseudomonal agents with a comparable antimicrobial spectrum, rather, their use also declined. These findings suggest that a simple substitution with alternative broad-spectrum agents did not occur. Consequently, the commonly described “domino effect,” wherein shortages of one antimicrobial agent precipitate increased use and subsequent shortages of alternatives, was effectively mitigated [15]. Furthermore, the increased use of narrow-spectrum agents likely reflects appropriate de-escalation practices, particularly because approximately 70% of ASP recommendations were accepted. Similarly, the observed increases in anti-MRSA agents, total intravenous antimicrobials, and combined intravenous and oral antimicrobial consumption were considered clinically appropriate. The rise in intravenous antimicrobial use appears to represent adherence to recommended treatment durations rather than inappropriate or unnecessarily prolonged therapy. To further contextualize our findings, prior studies evaluating the consequences of antimicrobial shortages have reported quantifiable shifts in prescribing patterns and clinical outcomes. For example, during the cefepime shortage in Switzerland, increased use of alternative broad-spectrum antimicrobials was observed, and a decline in *P. aeruginosa* susceptibility rates was reported [15]. Similarly, the shortage of piperacillin–tazobactam at the University of Mississippi Medical Center led to increased use of alternative broad-spectrum antimicrobials and a subsequent rise in vancomycin-resistant enterococci [16]. Effective management of antimicrobial shortages requires timely communication and rigorous monitoring [17]. Therefore, increased frequency of AST conferences supported by ID specialists, proactive involvement of ID specialists in managing positive blood culture cases, and provision of formal ID consultations were considered instrumental in promoting appropriate antimicrobial use under conditions of constrained supply.

Notably, a modest reduction in CAR-DOTs was observed between October 2023 and February 2024, prior to the official commencement of the intervention in April 2024. On October 17, 2023, a hospital-wide announcement was issued regarding the unstable supply of meropenem, advising prescribers to minimize its use to a case-by-case basis under the “usage-restricted” policy. Although this measure did not constitute a formal stewardship intervention, it may have raised awareness among clinicians and encouraged informal prescribing restraint before the official initiation of the ASP supported by ID specialists. This institutional influence likely contributed to the early decline in CAR-DOTs, which the counterfactual model did not interpret as regular seasonal variation.

Shortages of pharmaceuticals, including antimicrobial agents, are associated with adverse clinical outcomes, including increased mortality rates and prolonged lengths of hospital stay [4]. However, in the present study, the intervention did not lead to any deterioration in key patient outcomes, e.g., in-hospital mortality or length of stay. Given that antimicrobial agents are not readily interchangeable but critical as a life-saving treatment, the absence of worsened clinical outcomes during multiple antimicrobial shortages is particularly noteworthy. These findings underscore the importance of specialist involvement in antimicrobial decision-making, especially under constrained conditions. The complexity of antimicrobial selection during supply disruptions highlights the essential role of ID specialists in supporting optimal clinical management in such challenging settings.

In our study, the incidence of CRPA significantly decreased. This finding is particularly notable given that antimicrobial shortages typically increase the risk of antimicrobial resistance and CDI since clinicians tend to shift toward alternative broad-spectrum agents [1,5]. The observed reduction in CRPA strongly suggests that the involvement of ID specialists played a pivotal role in preventing such harmful shifts by ensuring judicious antimicrobial selection and promoting de-escalation strategies even under constrained supply conditions. These findings underscore the urgent need for strategic measures to prevent antimicrobial shortages. They also highlight how ID specialist–led stewardship can serve as a critical safeguard against the emergence and spread of resistant organisms during these periods. Moreover, our findings demonstrate that, even under multiple antimicrobial supply constraints, ID specialist–led stewardship was able to achieve improvements in antimicrobial use and reductions in antimicrobial-resistant organisms—outcomes that align with the core objectives outlined in established ASP guidelines [18].

Although the cost associated with CAR use decreased, overall antimicrobial expenditure significantly increased. This increase likely reflects a shift toward more appropriate antimicrobial agents and adherence to optimal treatment durations, rather than inappropriate or unnecessary prescribing.

In the present study, a statistically significant increase was observed in the number of culture specimens submitted following the implementation of the intervention. Given that the AST conferences primarily followed a desk-based format, this increase in culture specimen submissions likely contributed to more informed and evidence-based decision-making during stewardship discussions. It is important to acknowledge that, during the intervention period, a shortage of blood culture bottles persisted for approximately 3 months, from July 4 to September 30, 2024. This limited supply may have led to a temporary decline in the number of positive blood culture results, potentially impacting the assessment of antimicrobial appropriateness and stewardship recommendations during that timeframe [19]. However, despite the shortage of blood culture bottles during the post-intervention period, the number of blood culture submissions increased, suggesting that our intervention exerted a particularly strong effect.

This study has some limitations. First, it was conducted at a single university-affiliated hospital in Japan, which may limit the generalizability of the findings. However, university hospitals are characterized by frequent personnel changes and healthcare professional rotations, including physicians. This factor is known to hinder the consistent implementation of ASPs [20]. The fact that this intervention led to a reduction in the use of CAR and antipseudomonal agents without adversely affecting patient outcomes in such a dynamic setting suggests that the involvement of ID specialists can exert a robust and reproducible impact, potentially extendable to other institutions. Second, the nature and extent of antimicrobial supply restrictions likely vary across institutions. Nevertheless, individualized guidance by ID specialists on the selection of antimicrobial therapy remains a highly resilient and adaptable strategy under such heterogeneous conditions. Finally, despite increased AST conference frequency during the post-intervention period, more intensive follow-up on a case-by-case basis, independent of the direct involvement of ID specialists, may have contributed to the observed effects. However, the expansion of AST conference activities could not have been feasible without the support and leadership of ID specialists. Larger multi-center studies are needed to validate these findings, enhance generalizability, and assess effectiveness of ID specialist-led interventions.

## Conclusions

The implementation of an ASP supported by ID specialists/consultations successfully led to a significant reduction in the use of CARs, three antipseudomonal agents, and incidence of CRPA, without compromising patient outcomes, even under conditions of severe antibiotic shortages. These results highlight the critical role of ID specialists in ensuring an effective delivery of robust ASPs, especially during periods of constrained antimicrobial supply. Additionally, they underscore the growing importance of ID specialists in navigating the increasingly complex landscape of modern clinical care. In this context, our findings suggest that the involvement of ID specialists can serve as a practical strategy for maintaining appropriate antimicrobial use during periods of drug shortages.

## Supporting information

S1 File Supplementary_data. (DOCX)

S2 File Dataset. (XLSX)

## Abbreviations

AS: antimicrobial stewardship
ASPs: antimicrobial stewardship programs
ID: infectious disease
IRB: Institutional Review Board
NCUEMC: Nagoya City University East Medical Center
AST: antimicrobial stewardship team
CARs: carbapenems
MRSA: methicillin-resistant Staphylococcus aureus
DOTs: days of therapy
CDIs: Clostridioides difficile infections
BSTS: Bayesian structural time-series
CrIs: credible intervals
CRPA: carbapenem-resistant Pseudomonas aeruginosa.
