# Supplementary material for: Impact of an infectious disease specialist-led antimicrobial stewardship program and consultations during multiple antimicrobial shortages: A bayesian structural time-series analysis
Source: PLoS One. 2026 Feb 2;21(2):e0340599. doi: 10.1371/journal.pone.0340599 (PMC12863545; doi:10.1371/journal.pone.0340599)
Supplement: S1 File — (DOCX) [file pone.0340599.s001.docx]

**Supporting information**

**Impact of an infectious disease specialist-led antimicrobial stewardship program and consultations during multiple antimicrobial shortages: A Bayesian structural time-series analysis**

**SMethods**

**Antimicrobial stewardship program (ASP)**

During weekdays, infectious disease (ID) specialists promptly notified the primary physician teams of positive blood culture results to facilitate appropriate empirical treatment. Conversely, on weekends, neither the laboratory nor the ID specialists communicated blood culture results, and such reporting to the primary teams was deferred until the next weekday. When *Staphylococcus aureus* or *Candida* species were identified, an ID consultation was recommended to enhance patient outcomes, with bedside evaluations conducted accordingly. All cases reviewed during the conferences were recorded in the electronic medical record system. The antimicrobial stewardship team (AST) members assessed the interventions using the ABCDEFHIT criteria, to qualitatively evaluate the appropriateness of targeted antimicrobial use based on culture results, clinical course, and treatment duration (**S1 Table**) [1,2]. Assessments were performed within 48 hours of therapy initiation or as determined by the AST, which involved de-escalation or discontinuation based on culture results or imaging recommendations, as well as dose adjustments following laboratory test results. Evaluation decisions were made through consensus among all AST members, and findings were categorized accordingly. When the AST recommended adjustments to antimicrobial therapy or additional culture testing due to insufficient initial assessment, feedback was provided to the primary physician teams on the audit day, either via telephone or medical record documentation. All patients subject to audit were monitored daily on weekdays until the targeted antimicrobial therapy was either completed or as specified by the AST. The AST proactively contacted the primary physician teams whenever intervention was needed. When discrepancies arose between AST recommendations and the primary team's management decisions, discussions were held to reach a consensus, with the final decision ultimately resting with the primary team. If difficulties in diagnosis or treatment planning were encountered during conference-based medical record reviews, bedside evaluations by ID specialists were initiated, with formal consultations proposed to the primary physician teams as necessary.

**Days of therapy (DOTs) for antipseudomonal agents**

The total DOTs per 100 patient-days per month were determined for three intravenous antipseudomonal agents: piperacillin–tazobactam, cefepime, and ceftolozane–tazobactam. These agents were selected owing to their broad-spectrum activity comparable to that of carbapenems (CARs).

**DOTs for narrow-spectrum antimicrobials**

Intravenous penicillin G, ampicillin, ampicillin/sulbactam, cefazolin, cefmetazole, and flomoxef were categorized as narrow-spectrum antimicrobials. The total DOTs per 100 patient-days per month were calculated for these agents. These antimicrobials were evaluated as reference indicators for de-escalation from broad-spectrum therapy.

**DOTs for anti-methicillin-resistant *Staphylococcus aureus* (MRSA) antimicrobials**

Intravenous vancomycin, teicoplanin, daptomycin, and linezolid were classified as anti-MRSA agents, and their total DOTs per 100 patient-days per month were calculated.

**DOTs for all intravenous antimicrobials, all oral antimicrobials, and the combined total of intravenous and oral antimicrobials**

The total DOTs per 100 patient-days per month was determined for all intravenous antimicrobials, all oral antimicrobials, and the combined total of both routes. These measures were used to assess the overall impact of the intervention on antimicrobial utilization in hospitalized patients.

**Incidence of hospital-acquired resistant microorganisms and *Clostridioides difficile* infection (CDI)**

The incidence rates per 1000 patient-days of hospital-acquired resistant microorganisms and CDI were evaluated as the outcome measures of the ASP. The resistant microorganisms included carbapenemase-producing Enterobacterales (CPE), carbapenem-resistant Enterobacterales (CRE), carbapenem-resistance *Pseudomonas aeruginosa* (CRPA), MRSA, and extended-spectrum beta-lactamase (ESBL)-producing Enterobacterales. Hospital-acquired organisms were defined as those isolated more than 72 hours after admission [3]. To prevent duplication, only the first isolate per patient per month was considered [3]. However, a new event was defined when a resistant organism was isolated from the blood when the same organism had not been previously isolated within the preceding 2 weeks [3]. CDI was diagnosed when toxin production was detected using the C. DIFF QUIK CHEK COMPLETE test (Alere Medical Co., Tokyo, Japan) or nucleic acid amplification tests for *C. difficile* toxin genes using Xpert *C. difficile* (Cepheid, Sunnyvale, CA, USA). ESBL-producing Enterobacterales was identified using the disc diffusion method with cefotaxime/clavulanic acid or ceftazidime/clavulanic acid discs (Eiken Chemical Co., Ltd., Tokyo, Japan). CPEs were detected using the modified CAR inactivation method according to the Clinical and Laboratory Standards Institute (CLSI) M100-S27 guidelines [4]. CRE was defined as resistance to meropenem (minimum inhibitory concentration [MIC] ≥2 μg/mL) or resistance to imipenem (MIC ≥2 μg/mL) and cefmetazole (MIC ≥64 μg/mL) [5]. CRPA was defined as Pseudomonas aeruginosa exhibiting resistance to CARs as well as either aminoglycosides or fluoroquinolones according to CLSI antimicrobial susceptibility testing standards, irrespective of carbapenemase production [6].

**Cost of CARs and all antimicrobials**

The cost of antimicrobials per patient-day was calculated to assess the economic impact of the ASP. An exchange rate of 150 Japanese yen per 1 US dollar (as of March 2025) was applied.

**Number of each type of culture sample per 1000 patient-days of hospitalization**

The number of inpatient culture samples per 1000 patient-days was assessed to evaluate the influence of ID consultation and ASP implementation. Specimen types included blood, respiratory tract, gastrointestinal tract, genitourinary tract, puncture fluids, and others. Respiratory specimens included sputum, pharyngeal and nasal secretions, oral secretions, lung tissue, and bronchial lavage fluid. Gastrointestinal specimens comprised stool, bile, and pancreatic fluid. Genitourinary specimens included urine and vaginal secretions. Puncture fluids encompassed pleural, ascitic, cerebrospinal, synovial, and bone marrow fluids. Other specimens included catheter tips, wound cultures, drain cultures, and samples from miscellaneous sources.

**All-cause in-hospital mortality rate and length of hospital stay**

The 30-day all-cause in-hospital mortality rate and the length of hospital stay were analyzed to evaluate the clinical impact of ID consultation and ASP implementation.

**Assessment and acceptance rate of AST recommendations**

The AST evaluated the appropriateness of antimicrobial use and calculated the acceptance rate of recommendations, which was defined as the number of fully or partially accepted interventions divided by the total number of interventions. Evaluations classified as A or B were considered appropriate.

**S1 Table.** **Assessment sheet used by the AST**

|  | **Category** | | |
| --- | --- | --- | --- |
| **Appropriate therapy** | A | Appropriate | Antimicrobial selection and dosage are appropriate at the time of evaluation. |
|  | B | Better choice | There are no major problems with antimicrobial selection, although there are suggestions for some modifications and changes. |
| **Inappropriate therapy** | C | Culture | Absence or inadequacy of submission of bacterial cultures; requires additional investigation (or, therefore, is difficult to evaluate). |
|  | D | De-escalation | Broad-spectrum antimicrobials were used on the basis of the clinical characteristics, culture results, and local factors, and these can be changed to a narrow-spectrum antimicrobial. |
|  | E | Escalation | The antimicrobials do not provide adequate coverage of the target microorganisms; therefore, the spectrum needs to be broadened, or the antimicrobial should be changed. |
|  | F | Fitting dose | The dose and method of administering the antimicrobials are inappropriate based on renal function or other factors; thus, adjustments are necessary. |
|  | H | Halt | Discontinuation is necessary because the purpose of antimicrobial administration has been achieved, further use is unnecessary, or there is a risk of allergy. |
|  | I | Indication document | The purpose of use and the target microorganisms of the antimicrobials are not described in the medical record and, therefore, cannot be evaluated; additional descriptions are needed. |
| **Time out** | T | Time out | Notify physician that culture results are available (3–5 days after initiation of the antimicrobial therapy) or that it is time to reconsider the termination of antimicrobial therapy (10–14 days after initiation of the antimicrobial therapy). |

Abbreviation: AST, antimicrobial stewardship team

**References**

1. Itoh N, Akazawa-Kai N, Yamaguchi M, Kawabata T. Efficiency of a long-term infectious diseases consultation and antimicrobial stewardship program at a Japanese cancer center: an interrupted time-series analysis. Open Forum Infect Dis. 2024;11: ofae678. doi: [10.1093/ofid/ofae678](https://doi.org/10.1093/ofid/ofae678).

2. Itoh N, Akazawa N, Kanawaku E, Murakami H, Ishibana Y, Kawamura D, et al. Effects of infectious disease consultation and antimicrobial stewardship program at a Japanese cancer center: an interrupted time-series analysis. PLOS One. 2022;17: e0263095. doi: [10.1371/journal.pone.0263095](https://doi.org/10.1371/journal.pone.0263095).

3. Centers for Disease Control and Prevention. Multidrug-resistant organism and Clostridioides difficile infection (MDRO/CDI) module. Available from: https://www.cdc.gov/nhsn/PDFs/pscManual/12pscMDRO_CDADcurrent.pdf

4. Clinical and Laboratory Standards Institute (CLSI). Performance standards for antimicrobial susceptibility testing. CLSI supplement M100-S27. 27th ed. Wayne: CLSI; 2017.

5. Yonekawa S, Mizuno T, Nakano R, Nakano A, Suzuki Y, Asada T, et al. Molecular and epidemiological characteristics of carbapenemase-producing Klebsiella pneumoniae clinical isolates in Japan. mSphere. 2020;5: e00490-20. doi: [10.1128/mSphere.00490-20](https://doi.org/10.1128/msphere.00490-20).

6. Akazawa T, Kusama Y, Fukuda H, Hayakawa K, Kutsuna S, Moriyama Y, et al. Eight-year experience of antimicrobial stewardship program and the trend of carbapenem use at a tertiary acute-care hospital in Japan-the impact of postprescription review and feedback. Open Forum Infect Dis. 2019;6: ofz389. doi: [10.1093/ofid/ofz389](https://doi.org/10.1093/ofid/ofz389).
